# Predictive Performance of the Simplified Acute Physiology Score (SAPS) II and the Initial Sequential Organ Failure Assessment (SOFA) Score in Acutely Ill Intensive Care Patients: Post-Hoc Analyses of the SUP-ICU Inception Cohort Study

**DOI:** 10.1371/journal.pone.0168948

**Published:** 2016-12-22

**Authors:** Anders Granholm, Morten Hylander Møller, Mette Krag, Anders Perner, Peter Buhl Hjortrup

**Affiliations:** Department of Intensive Care, Copenhagen University Hospital – Rigshospitalet, Copenhagen, Denmark; Johns Hopkins University Bloomberg School of Public Health, UNITED STATES

## Abstract

**Purpose:**

Severity scores including the Simplified Acute Physiology Score (SAPS) II and the Sequential Organ Failure Assessment (SOFA) score are used in intensive care units (ICUs) to assess disease severity, predict mortality and in research. We aimed to assess the predictive performance of SAPS II and the initial SOFA score for in-hospital and 90-day mortality in a contemporary international cohort.

**Methods:**

This was a post-hoc study of the Stress Ulcer Prophylaxis in the Intensive Care Unit (SUP-ICU) inception cohort study, which included acutely ill adults from ICUs across 11 countries (n = 1034). We compared the discrimination of SAPS II and initial SOFA scores, compared the discrimination of SAPS II in our cohort with the original cohort, assessed the calibration of SAPS II customised to our cohort, and compared the discrimination for 90-day mortality vs. in-hospital mortality for both scores. Discrimination was evaluated using areas under the receiver operating characteristics curves (AUROC). Calibration was evaluated using Hosmer-Lemeshow’s goodness-of-fit Ĉ-statistic.

**Results:**

AUROC for in-hospital mortality was 0.80 (95% confidence interval (CI) 0.77–0.83) for SAPS II and 0.73 (95% CI 0.69–0.76) for initial SOFA score (P<0.001 for the comparison). Calibration of the customised SAPS II for predicting in-hospital mortality was adequate (P = 0.60). Discrimination of SAPS II was reduced compared with the original SAPS II validation sample (AUROC 0.80 vs. 0.86; P = 0.001). AUROC for 90-day mortality was 0.79 (95% CI 0.76–0.82; P = 0.74 for comparison with in-hospital mortality) for SAPS II and 0.71 (95% CI 0.68–0.75; P = 0.66 for comparison with in-hospital mortality) for the initial SOFA score.

**Conclusions:**

The predictive performance of SAPS II was similar for in-hospital and 90-day mortality and superior to that of the initial SOFA score, but SAPS II’s performance has decreased over time. Use of a contemporary severity score with improved predictive performance may be of value.

## Introduction

Severity scoring systems are frequently used in intensive care units (ICUs) to assess disease severity, predict mortality, compare ICU performances, and in research [1–3]. The scores generally belong to one of two groups: 1) scores that aim to predict mortality based on parameters obtained upon ICU admission or during the first 24 hours of ICU stay, or 2) scores that aim to quantify the level of organ dysfunction daily during ICU stay [1–3].

The Simplified Acute Physiology Score (SAPS) II was developed and validated in a European and North American cohort (n = 12,997), and published in 1993 [4]. The score includes 17 variables collected during the first 24 hours of ICU stay. Based on the sum of the score the in-hospital mortality risk can be estimated [4].

The Sequential Organ-Failure Assessment (SOFA) score was developed by an expert panel in 1996 [5]. The worst values recorded for every 24-hour period in the ICU is used to assign a score of 0–4 for six organ systems. Although the score was developed to describe changes in organ dysfunction throughout ICU stay, and not to predict mortality, an association between increasing initial organ-specific SOFA scores and mortality has been suggested [5].

The predictive performance of SAPS II has been evaluated in multiple studies, generally showing acceptable discrimination but poor calibration in other populations than the original one [6–15]. In a systematic review, Minne et al. found that the initial SOFA score showed good to excellent discrimination between subsequent hospital survivors and non-survivors, with an area under the receiver operating characteristic curve (AUROC) similar to that of SAPS II [16]. The authors concluded that the SOFA score performed similarly to the SAPS II, however their systematic review included only one small single-centre study directly comparing the two scores, and this needs to be further assessed in larger multi-centre studies.

The value of the mortality prediction scores appear to deteriorate over time [13, 17]. SAPS II has been re-assessed in several national studies [6–15], but its performance is affected by case-mix [2, 18] and national differences. Performance in multinational, contemporary cohorts has, however, been less studied. Additionally, SAPS II was developed to predict in-hospital mortality. In-hospital mortality is considered an inadequate outcome measure today, as it is affected by hospital discharge practice [19–21]. Use of a long-term, fixed-time mortality endpoint might affect the predictive performance of these scores.

The aim of this study was to assess the predictive performances of SAPS II and the initial SOFA score for in-hospital mortality vs. 90-day mortality in a contemporary international general ICU cohort of acutely ill adults. We hypothesised that the predictive performance of SAPS II and the initial SOFA score for in-hospital mortality in ICUs today would be low, and that the predictive performance for 90-day mortality would be superior to that for in-hospital mortality.

## Methods

This study was a post-hoc study of the Stress Ulcer Prophylaxis in the Intensive Care Unit (SUP-ICU) 7-day international inception cohort study [22], in which all acutely admitted patients aged 18 year or above were included in 97 ICUs in 11 countries during a single 7-day period between 1st December 2013 and 30th April 2014. Exclusion criteria were gastrointestinal (GI) bleeding upon ICU admission and previous ICU admission during the index hospital admission. The SUP-ICU cohort study was approved by the Danish Data Protection Agency (No. 30–1115) and the Danish Health and Medicines Authorities (No. 3-3013-463/1/). Ethical approval was not applicable owing to the non-interventional (observational) design. Patient information in the SUP-ICU cohort study database was anonymised and de-identified.

For the present study, an internal protocol and statistical analysis plan was written prior to the analyses. We included all the 1034 patients in the SUP-ICU cohort study database and extracted the following data: Age, gender, SAPS II, initial SOFA score, comorbidities, interventions given at ICU admission, in-hospital mortality and 90-day mortality. The present manuscript was prepared according to the Strengthening the Reporting of Observational Studies in Epidemiology (STROBE) Statement [23].

### Registration of SAPS II and initial SOFA scores

Data were registered prospectively using a secure web-based electronic case report form, as previously reported [22]. Data for the SAPS II and initial SOFA scores were recorded during the first 24 hours of ICU admission, as defined in the original scores [4, 5]. The Glasgow Coma Scale (GCS) score was registered in accordance with the SAPS II definition, i.e. if patients were sedated, the last known value before sedation was recorded and used for both the SAPS II and the SOFA score.

### Measures of predictive performance

For the evaluation of the predictive performances of the scores, we primarily focused on the discrimination of the scores. Discrimination is the ability of the score to separate patients that die from patients that live, and discrimination was assessed using AUROCs as described in the next section.

We compared the discrimination of the SAPS II and initial SOFA scores, and compared the discrimination of SAPS II in the present cohort with the original cohort. Additionally, we customised SAPS II by re-calibrating it to our cohort and assessed calibration (the prognostic accuracy of the model at different risk intervals, described in the next section) of the customised SAPS II. We did this to assess the association between SAPS II and mortality in the original cohort and in the present cohort.

Finally, we compared the discrimination when using 90-day vs. in-hospital mortality as the outcome measure for both the SAPS II and the initial SOFA score

### Statistical analysis

We did all statistical analyses according to the analysis plan using SAS version 9.3 (SAS Institute Inc., Cary, NC, USA). As this was a post-hoc analysis of the SUP-ICU cohort study, a convenience sample was used and thus no sample size calculation was made.

We stratified patient characteristics on inclusion in the SUP-ICU cohort study by 90-day mortality and presented these as medians with interquartile ranges (IQR) for continuous data, and numbers (%) for categorical data [24]. We assessed differences by Mann–Whitney U test and Chi^2^-test. All statistical tests were two-tailed, and P-values < 0.05 were considered statistically significant.

We knew variables were missing in the SAPS II and initial SOFA scores [22], and as reported in the main SUP-ICU cohort publication, data were not *missing completely at random* (Little’s test, P <0.001) [22]; thus multiple imputations for the missing SAPS II and SOFA scores were performed [25, 26]. Multiple imputations were performed using the fully conditional specification method and 10 imputed datasets. For patients with missing data, the complete scores were imputed, not their individual components, based on the following baseline variables: age; gender; SAPS II/initial SOFA score; chronic obstructive pulmonary disease, asthma or chronic lung disease; previous myocardial infarction; severe chronic heart failure (New York Heart Association Functional Classification class 3–4); chronic renal failure; liver cirrhosis or increased bilirubin; metastatic cancer; active haematological cancer; AIDS; mechanical ventilation at ICU admission; circulatory support at ICU admission; and renal replacement therapy at ICU admission. The discrimination of the two scores (the ability of the model to separate patients who dies from patients who lives) was assessed by their AUROCs. Comparison of differences in the AUROCs between SAPS II and the initial SOFA score according to in-hospital mortality was performed as described by DeLong et al [27]. The method by DeLong et al. is a non-parametric method for comparing different AUROCs, based on the theory of generalised U-statistics that generates a covariance matrix and produces a test-statistic with a Chi^2^-distribution [27].

The standard error of the AUROC in the original SAPS II validation sample [4] was calculated from the 95% confidence interval [28] and used for the comparison. We used Chi^2^-test to compare the differences in AUROCs between SAPS II for in-hospital mortality in the original SAPS II validation sample and in our cohort and to compare the differences in AUROC between in-hospital vs. 90-day mortality for SAPS II and the initial SOFA score in our cohort [29].

We performed a first-level customisation of SAPS II for in-hospital mortality in the SUP-ICU cohort, as the original SAPS II equation has already been reported to have deteriorated since its development [6–15]. In a first-level customisation, logistic regression analysis is used to derive a new equation while the variables included in the score and their weights are left unchanged.

The calibration (the prognostic accuracy of the model at different risk intervals) of the customised SAPS II was evaluated using the Hosmer-Lemeshow goodness-of-fit Ĉ-statistic.

A post-hoc sensitivity analysis of complete cases only (instead of the multiple imputated dataset) was conducted during the peer-review process (S1 Appendix).

## Results

All 1034 patients were included; in-hospital and 90-day mortality rates were 22.5% (95% confidence interval (CI) 20.0–25.2; 233/1027, 7 missing values) and 26.2% (95% CI 23.6–29.0; 271/1034), respectively.

One or more of the variables in the SAPS II and initial SOFA scores were missing for 17.4% (n = 180) and 23.4% (n = 245) of the included patients, respectively [22]. Thus, for 17.4% of the patients, multiple imputations were made for the full SAPS II scores, and for 23.4% of the patients, multiple imputations were made for the full initial SOFA scores.

Patients who died within 90 days were older, had more comorbidities, and higher SAPS II and initial SOFA scores than those who survived (Table 1).

**Table 1:** Patient characteristics at ICU admission.

Characteristic	All (n = 1034)	Alive 90 days after ICU admission (n = 763)	Dead 90 days after ICU admission (n = 271)	P-value*	Patients with missing values, n (%)
Age—years—median (IQR)	63 (48–74)	60 (45–71)	71 (60–79)	< 0.001	0 (0.0)
Male gender—no. (%)	576 (55.7)	421 (55.2)	155 (57.2)	0.57	0 (0.0)
Initial SOFA score—median (IQR)	6 (4–8)	6 (3–7)	8 (5–11)	< 0.001	245 (23.4)
SAPS II—median (IQR)	42 (31–54)	37 (28–48)	57 (47–68)	< 0.001	180 (17.4)
Chronic obstructive pulmonary disease, asthma or other chronic lung disease—no. (%)	205 (19.8)	138 (18.1)	67 (24.7)	0.02	0 (0.0)
Previous myocardial infarction—no. (%)	103 (10.0)	63 (8.3)	40 (14.8)	0.002	0 (0.0)
Severe chronic heart failure (NYHA 3–4)—no. (%)	56 (5.4)	22 (2.9)	34 (12.6)	< 0.001	0 (0.0)
Chronic renal failure—no. (%)	74 (7.2)	45 (5.9)	29 (10.7)	0.008	0 (0.0)
Liver cirrhosis or increased bilirubin (> 33 μmol/l)—no. (%)	124 (12.5)	79 (10.8)	45 (17.2)	0.007	38 (3.7)
Metastatic cancer—no. (%)	46 (4.5)	23 (3.0)	23 (8.5)	< 0.001	0 (0.0)
Active hematologic cancer—no. (%)	36 (3.5)	17 (2.2)	19 (7.0)	< 0.001	0 (0.0)
AIDS—no. (%)	3 (0.3)	2 (0.3)	1 (0.4)	1.0	0 (0.0)
Immunosuppression**—no. (%)	50 (4.8)	33 (4.3)	17 (6.3)	0.20	0 (0.0)
Coagulopathy on ICU admission***—no. (%)	128 (12.4)	68 (8.9)	60 (22.1)	< 0.001	0 (0.0)
Comorbidities—no. (%)					
0	501 (48.5)	414 (54.3)	87 (32.1)	< 0.001	0 (0.0)
1	318 (30.8)	232 (30.4)	86 (31.7)	0.68	0 (0.0)
2	153 (14.8)	96 (12.6)	57 (21.0)	< 0.001	0 (0.0)
3	46 (4.5)	16 (2.1)	30 (11.1)	< 0.001	0 (0.0)
> 3	16 (1.6)	5 (0.7)	11 (4.1)	< 0.001	0 (0.0)
Mechanical ventilation on ICU admission—no. (%)	544 (52.6)	377 (49.4)	167 (61.6)	< 0.001	0 (0.0)
Circulatory support on ICU admission—no. (%)	469 (45.7)	293 (38.6)	176 (65.7)	< 0.001	7 (0.7)
Renal replacement therapy on ICU admission—no. (%)	70 (6.8)	36 (4.7)	34 (12.6)	< 0.001	0 (0.0)

* For the comparison of patients stratified by 90-day mortality.

** Defined as treatment with at least 0.3 mg/kg/day of prednisolone equivalent for one month or longer in the 6 months prior to ICU admission.

*** Platelets < 50 * 10^9^/l (50,000 mm^3^) and/or International Normalised Ratio (INR) > 1.5 during current hospital admission.

IQR: Interquartile range; NYHA: New York Heart Association Functional Classification; AIDS: Acquired Immune Deficiency Syndrome.

### SAPS II vs. initial SOFA score

**Fig 1:**
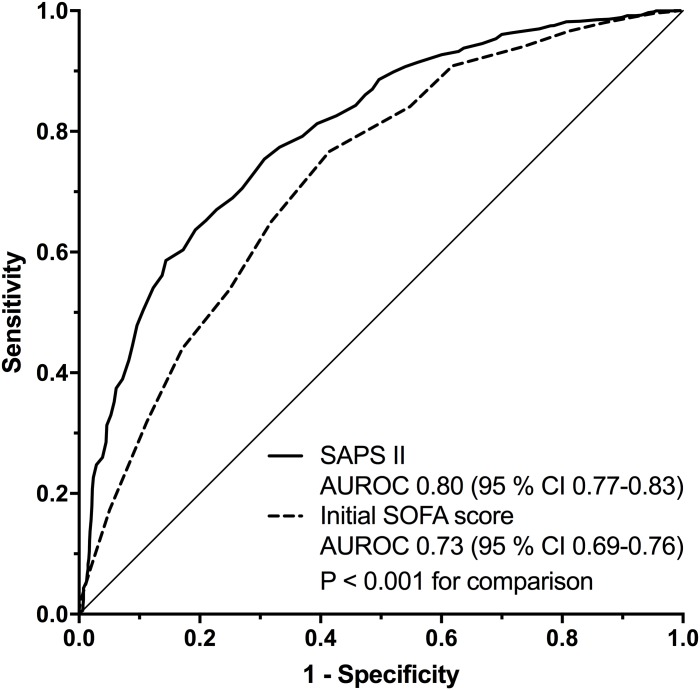
Discrimination of SAPS II and the initial SOFA score for in-hospital mortality. Receiver operating characteristics curves for SAPS II and the initial SOFA score for in-hospital mortality. AUROC: Area under the receiver operating characteristics curve.

The ROC curves for SAPS II and the initial SOFA score for in-hospital mortality are shown in Fig 1. The AUROCs for SAPS II was higher than that for initial SOFA scores for in-hospital mortality; 0.80 (95% CI 0.77–0.83) and 0.73 (95% CI 0.69–0.76), respectively (P < 0.001).

### SAPS II in the present cohort vs. SAPS II in the original cohort

Discrimination of SAPS II for in-hospital mortality in the present cohort was lower than that of the original SAPS II validation sample (AUROC 0.80 vs. AUROC 0.86; P = 0.001).

**Fig 2:**
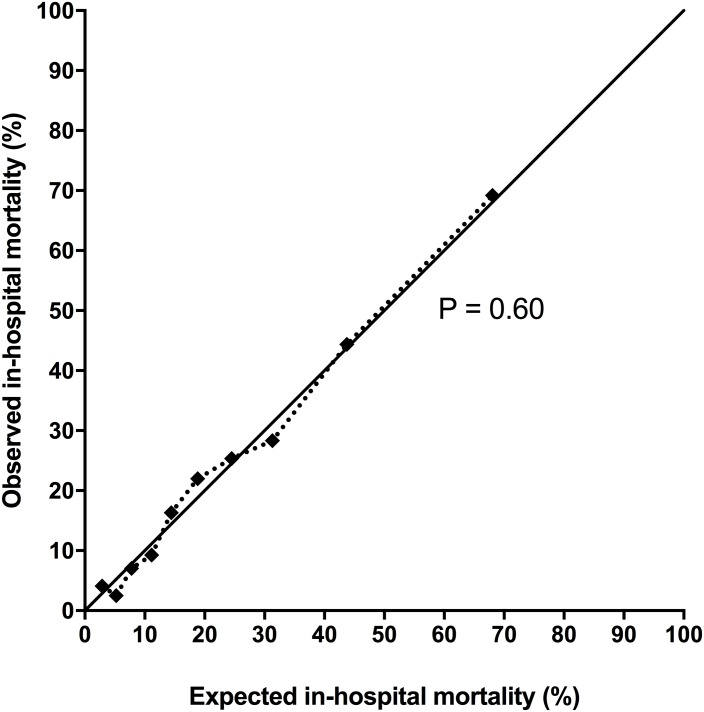
Calibration of the customised SAPS II for in-hospital mortality. Calibration curve of SAPS II customised by logistic regression analysis for in-hospital mortality and assessed by the Hosmer-Lemeshow goodness-of fit Ĉ-statistic. The full line included on the figure is the line of equality (expected = observed mortality).

**Fig 3:**
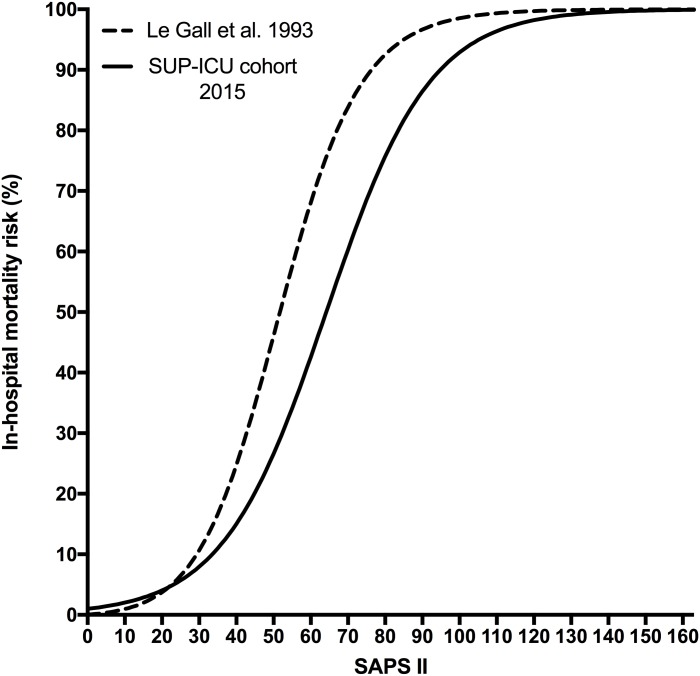
SAPS II and in-hospital mortality risk in the original and the present cohort. Correlation between SAPS II scores and in-hospital mortality risk in the original and the present cohort. In-hospital mortality risks are according to the original SAPS II equation (Le Gall et al. 1993) and the equation of SAPS II re-calibrated in the present cohort.

Calibration of the customised SAPS II was good (Fig 2). The correlation between SAPS II and in-hospital mortality risk in our cohort and the original SAPS II validation sample is shown in Fig 3 and Table 2.

**Table 2:** SAPS II and in-hospital mortality risk in the original and the present cohort.

SAPS II score	Le Gall et al. 1993	SUP-ICU cohort 2015
10	1.0%	2.0%
20	3.7%	4.0%
30	10.6%	7.9%
40	24.7%	15.0%
50	46.1%	26.6%
60	68.1%	42.6%
70	83.8%	60.3%
80	92.5%	75.7%
90	96.7%	86.4%
100	98.5%	92.9%

Correlation between SAPS II scores and in-hospital mortality risk in the original and the present cohort for selected SAPS II scores.

### In-hospital vs. 90-day mortality

**Fig 4:**
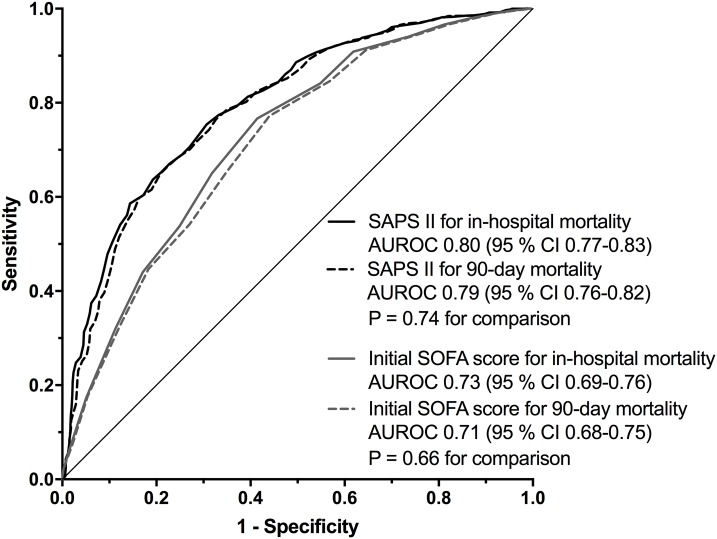
Discrimination of SAPS II and the initial SOFA score for in-hospital and 90-day mortality. Receiver operating characteristics curves for SAPS II and the initial SOFA score for in-hospital and 90-day mortality. AUROC: Area under the receiver operating characteristics curve.

The ROC curves for SAPS II and initial SOFA scores for in-hospital and 90-day mortality are shown in Fig 4. For SAPS II the AUROCs were 0.80 (95% CI 0.77–0.83) and 0.79 (95% CI 0.76–0.82) for in-hospital mortality and 90-day mortality, respectively (P = 0.74 for the comparison). For the initial SOFA score the AUROCs were 0.73 (95% CI 0.69–0.76) and 0.71 (95% CI 0.68–0.75) for in-hospital mortality and 90-day mortality, respectively (P = 0.66 for the comparison).

The results of the post-hoc sensitivity analysis of the complete cases were consistent with the primary analysis of the imputed dataset (S1 Appendix).

## Discussion

In this study of the performances of SAPS II and the initial SOFA score in a contemporary cohort of general ICU patients, we found that 1) the discrimination of SAPS II was superior to that of the initial SOFA score for in-hospital mortality, 2) the predictive performance of SAPS II was lower in our cohort than that in the original cohort, and 3) the discriminative powers of the two scores were comparable for in-hospital mortality and 90-day mortality.

### SAPS II vs. the initial SOFA score

The predictive performance of the initial SOFA score has been assessed in a systematic review from 2008 by Minne et al. They summarised AUROCs of the initial SOFA score in 12 studies and concluded that the initial SOFA score was not inferior to SAPS II [16], although their review only included a single study that directly compared the two scores and this was in a small, selected patient population from a single ICU [30]. The discrimination of the initial SOFA score in our study was within the range reported by Minne et al., but inferior when compared with that of SAPS II. Importantly, the SOFA score was not developed to predict mortality, but to assess organ failure over time [5].

### SAPS II in the present cohort vs. SAPS II in the original cohort

Studies revisiting the performance of SAPS II have generally found good discrimination but poor calibration of the original equation [6–15]. Due to this, expected mortality ratios calculated using the original SAPS II equation in contemporary ICU patients are imprecise and in general too high, which is supported by the different correlations between SAPS II and in-hospital mortality in the original study vs. the present study. The imprecise observed/expected mortality ratios are likely caused by advances in intensive care, as the in-hospital mortality rate for ICU patients has declined over time [31–34], despite increasing age and severity of illness [31, 34]. Case-mix is known to affect the predictive powers of severity scores [18], however, even after adjustment for risk factors and case-mix, in-hospital mortality for patients admitted to the ICU has declined over time [32–34]. Accordingly, SAPS II has been re-calibrated in multiple studies improving the predictive performance [6, 8, 10, 12, 13, 15].

Our results show that not only the calibration of SAPS II is affected. The discrimination of the score has decreased slightly when used in contemporary ICU patients compared to the original study, and this decrease was statistically significant. While first-level customisations are simple to conduct and generally lead to adequate calibration of SAPS II—as was seen in the present study—first-level customisations do not increase discrimination, as the individual parameters and weights of the score are unchanged.

The somewhat inadequate predictive performance of SAPS II in contemporary general ICU patients has intrigued refinement of the score by including additional variables [12], and in 2005 the SAPS 3 score was developed [35, 36]. When compared with SAPS II, SAPS 3 has not been shown to perform markedly better [37–41], which may be why SAPS II is still widely used, despite its limitations.

### In-hospital vs. 90-day mortality

The AUROCs for SAPS II and the initial SOFA score for in-hospital vs. 90-day mortality were not different. Several studies have demonstrated that the performance of scores that rely solely on data collected during the first 24 hours of ICU stay (SAPS, SAPS II and the initial SOFA score) decrease with increasing lengths of stay [15, 42–44]. It has also been argued that the scores predict the obvious [43, 45]; patients presenting with the worst conditions will have the most deranged physiological parameters within the first 24 hours, causing high scores and high predicted as well as observed mortality rates. This may explain why 90-day mortality, though generally considered a more adequate outcome measure than in-hospital mortality [19–21], did not lead to improved predictive performance of SAPS II and initial SOFA scores in our study.

### Limitations with the use of SAPS II and initial SOFA scores for mortality prediction today

As highlighted above and in previous studies, the ability of SAPS II and initial SOFA scores to predict mortality has limitations, as the SOFA score was not developed to predict mortality and the predictive performance of the SAPS II has deteriorated. This may limit their clinical use in contemporary ICU cohorts in research and in benchmarking using standardised mortality ratios, unless the scores are regularly re-calibrated. Other potential challenges with the use of SAPS II and the initial SOFA score exist. First, missing values for some variables often occur (e.g. bilirubin and urea levels) [46, 47], which may affect the sum of the scores, and hence result in inadequate prediction. Second, the quality of treatment in the ICU can affect the scores and mask the true baseline differences, due to the use of the worst recorded parameters over a relatively long period of 24 hours [48]. Third, if the scores are used as originally proposed (worst values recorded during first 24 hours in the ICU), there is a risk of intervention effect on the scores in randomised clinical trials, which also may affect the predictive performance. This is often avoided by using values recorded within the 24 hours prior to, instead of after, inclusion in the trial [46, 47]. Finally, both scores include the GCS score, which poses a challenge when assessing sedated and mechanically ventilated patients. Of note, with SAPS II the GCS before sedation should be used [4], while the actual or assumed GCS should be used in the SOFA score [5]; this may result in discrepancies between the two scores.

When severity scores are used to describe populations in ICU studies, we believe that using both SAPS II and SOFA scores and additionally reporting age, important comorbidities and vital signs is appropriate. Use of severity scores that include variables readily available upon ICU admission may also prove to be of value.

### Strengths and limitations

The strengths of this study includes high generalisability (external validity), as contemporary ICU patients from 97 ICUs in 11 countries were included [22]. Second, data were prospectively registered in an electronic case report form, limiting the risk of information and selection bias, and third, the protocol and statistical analysis plan was prepared prior to analysis, reducing the risk of selection bias and data-driven analyses. Our study comes with limitations as well. First, patients suffering from GI bleeding upon admission to the ICU were excluded from the SUP-ICU cohort study, and thus the results from our study cannot be generalised to patients with GI bleeding admitted to the ICU. Second, SAPS II and initial SOFA scores were missing for 17.4% and 23.4% of the patients in the SUP-ICU database, respectively. We addressed this issue as recently recommended by using multiple imputations, as complete case analysis can bias the results [25]. The results of our sensitivity analysis of the complete case dataset were similar to the analysis of the imputed dataset. Third, this was a post-hoc study, which generally increases the risk for selection bias and spurious findings. Fourth, the sample used in this study was relatively small compared to the original development samples and several retrospective studies evaluating the scores. Finally, the participating ICUs may not be representative of ICUs in other countries.

## Conclusion

In this post-hoc study we assessed the performances of SAPS II and the initial SOFA score in contemporary ICU patients. We found that discrimination of SAPS II was superior to that of the initial SOFA score for in-hospital mortality, the predictive performance of SAPS II has decreased over time, and the discriminative powers of the two scores were not affected by extending the observation period from in-hospital mortality to 90-day mortality.

Consequently, the use of SAPS II and the initial SOFA score for mortality prediction purposes in contemporary ICU patients is not without limitations, and the development of a contemporary mortality prediction score may be of value.

## Supporting Information

S1 Appendix Sensitivity analysis using complete case analyses. (DOCX)

S1 Dataset Dataset SAS7BDAT. (SAS7BDAT)
