# Supplementary material for: Predictive Performance of the Simplified Acute Physiology Score (SAPS) II and the Initial Sequential Organ Failure Assessment (SOFA) Score in Acutely Ill Intensive Care Patients: Post-Hoc Analyses of the SUP-ICU Inception Cohort Study
Source: PLoS One. 2016 Dec 22;11(12):e0168948. doi: 10.1371/journal.pone.0168948 (PMC5179262; doi:10.1371/journal.pone.0168948)
Supplement: S1 Appendix — (DOCX) [file pone.0168948.s001.docx]

**S1 Appendix: Sensitivity analyses using complete case analysis**

This is a supplementary file to the paper: “Predictive Performance of the Simplified Acute Physiology Score (SAPS) II and Initial Sequential Organ Failure Assessment (SOFA) Score in Acutely Ill Intensive Care Patients: Post-Hoc Analyses of the SUP-ICU Inception Cohort Study”, by Granholm A, Møller MH, Krag M, Perner A and Hjortrup PB.

Due to missing data, all analyses in the main paper are performed using multiple imputations. As a sensitivity analysis, all analyses are repeated in this appendix using complete case analysis.

AUROC: Area under the receiver operating characteristics curve; CI: Confidence interval

**SAPS II vs. the initial SOFA score for in-hospital mortality**SAPS II AUROC: 0.81 (95 % CI 0.78 – 0.85)
Initial SOFA score AUROC: 0.72 (95 % CI 0.68 – 0.76)
Test for difference between AUROCs: P < 0.001

**SAPS II in the present cohort vs. SAPS II in the original cohort**Test for difference between AUROCs: P = 0.017
Test of calibration of SAPS II calibrated to in-hospital mortality in the SUP-ICU inception cohort (Hosmer-Lemeshow goodness-of-fit Ĉ-statistic): P = 0.91
Table: SAPS II and in-hospital mortality in the present cohort.

| **SAPS II score** | **Complete case analysis SUP-ICU cohort** |
| --- | --- |
| 10 | 1.4 % |
| 20 | 3.0 % |
| 30 | 6.6 % |
| 40 | 13.8 % |
| 50 | 26.6 % |
| 60 | 45.0 % |
| 70 | 64.9 % |
| 80 | 80.7 % |
| 90 | 90.4 % |
| 100 | 95.5 % |

**SAPS II for in-hospital vs. 90-day mortality**SAPS II AUROC for in-hospital mortality: 0.81 (95 % CI 0.78 – 0.85)
SAPS II AUROC for 90-day mortality: 0.80 (95 % CI 0.76 – 0.84)
Test for difference between AUROCs: P = 0.67

**Initial SOFA score for in-hospital vs. 90-day mortality**Initial SOFA score AUROC for in-hospital mortality: 0.72 (95 % CI 0.68 – 0.76)
Initial SOFA score AUROC for 90-day mortality: 0.71 (95 % CI 0.67 – 0.75)
Test for difference between AUROCs: P = 0.70
